# Case Series of Lipid Accumulation in the Human Corpus Cavernosum

**DOI:** 10.1097/MD.0000000000000550

**Published:** 2015-02-13

**Authors:** Amjad Alwaal, Lin Wang, Uwais B. Zaid, Guiting Lin, Tom F. Lue

**Affiliations:** From the Department of Urology (AA, LW, UBZ, GL, TFL), University of California, San Francisco, California; and King Abdulaziz University (AA), Jeddah, Saudi Arabia.

## Abstract

Erectile dysfunction is a prevalent problem affecting millions of men in the United States and around the world. There have been no reports of the presence of lipids within the human penile corporal bodies, whether in normal or diseased states. We present here a case series of 9 patients who underwent penile corporal tissue biopsy during penile prosthesis insertion with severe intracorporal fibrosis and difficulties during insertion.

Oil Red O staining was done to identify lipids; LipidTOX and phalloidin double staining was used to identify lipid location within the corpora, and Masson's trichrome staining was done to assess fibrosis.

We identified lipid accumulation in those 9 corporal tissue samples, and further analysis showed the distribution to be 10% intramyocellular lipids and 90% extramyocellular lipids. These 9 specimens contained increased amount of collagen when compared with controls. In addition, we analyzed corporal samples from 10 random erectile dysfunction patients presenting for penile prosthesis insertion and identified no lipid accumulation in those control patients.

This is the first report of lipid accumulation in the human corpus cavernosum. Possible mechanisms of lipid accumulation include androgen deficiency and dedifferentiation of corpus smooth muscle cells into other phenotypes; however, the exact mechanism is unknown and further research is needed.

## INTRODUCTION

Erectile dysfunction (ED) is a prevalent problem affecting millions of men in the United States and around the world.^1^ It is defined as the inability to achieve or maintain an erection satisfactory for sexual intercourse.^2^ There are several causes of ED, aging representing the most important factor.^3^ It has been reported that as many as 67% of men 70 years old are affected with ED.^4^ Other significant causes of ED include diabetes mellitus (DM), radical prostatectomy (RP), prostatic irradiation, and Peyronie's disease (PD).^3,5^ ED postpriapism represents a unique form of ED. After a major priapism episode that could last from several hours to several days, the normal corporal tissue is replaced with a dense fibrous scar that could render the patient impotent.^6^ ED management differs according to patient characteristics, severity of disease, and etiology. It ranges from lifestyle modifications such as diet control, exercise, and weight loss to surgical intervention with a penile prosthesis. Prior to surgical intervention, several treatment methods are available and can be offered to the patient, such as oral type 5 phosphodiesterase inhibitors, intracavernosal injections, intraurethral alprostadil, and vacuum erection device.^7^

The human penis is composed of a corpus spongiosum and 2 corpora cavernosal bodies. The 2 spongy corpora cavernosa are encased in a thick layer of fibrous tunica albuginea, which forms part of a fibrous skeleton that supports the corpora cavernosa. This also consists of an incomplete septum separating the 2 corporal bodies, fibrous intracavernosal pillars, intracavernous fibrous framework, and periarterial and perineural fibrous sheath. Within the corpora cavernosa, there is an extensive network of interconnected sinusoids separated by smooth muscle trabeculae and surrounded by collagen, elastic fibers, and loose areolar tissue. The terminal cavernous nerve fibers and helicine arteries are closely associated with the smooth muscles.^8,9^

To our knowledge, there have been no reports of the presence of lipids within the human penile corporal bodies, both in normal or diseased states, and no mention in previous studies of experiments done to look specifically for lipids in human penile tissue. We present the first case series of 9 patients who underwent penile corporal tissue biopsy during penile prosthesis surgery due to severe intracorporal fibrosis. We identified lipid accumulation in these 9 corporal tissue samples, which represents the first report of its kind.

## METHODS

### Case Series

We evaluated histological specimens from the corpus cavernosa of 9 patients who underwent surgery between January 2007 and July 2013. Specimens were taken intraoperatively during penile prosthesis insertion for ED. We specifically took specimens from patients in whom we encountered severe fibrotic changes in the corpora, which made penile prosthesis insertion difficult and required carving out the fibrotic intracorporal tissue prior to prosthesis insertion. Those corporal specimens were taken from the subtunical region near the proximal tunical incision. We decided to analyze those specimens and identify their histological changes, and examine them for the presence of lipids due to their shiny whitish appearance. Approval from the University of California, San Francisco, institutional review board was obtained for the study.

The patients’ ages ranged from 43 to 70 years (mean age 54.1 years), and their characteristics are listed in Table 1. In summary, 5 patients had hypertension (55.6%), 4 patients had history of priapism (44.4%), 3 patients had previously undergone failed penile prosthesis placement (33.3%), 2 patients had DM (type 2) (22.2%), 2 patients had PD (22.2%), 1 patient had undergone previous RP (11.1%), 1 patient had dyslipidemia (11.1%), and 1 patient had coronary artery disease (11.1%).

**TABLE 1:**
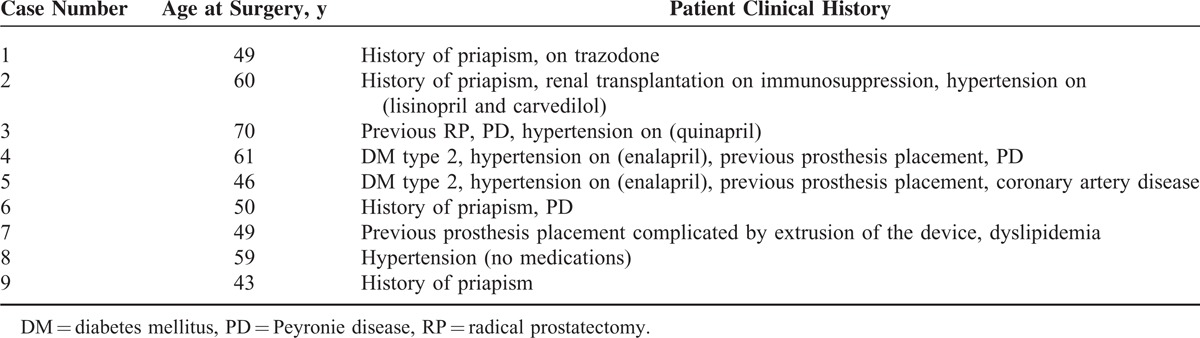
Characteristics of Patients With Intracorporal Lipids

Control specimens were taken from 10 random ED patients who presented for penile prosthesis insertion but did not have significant intracorporal fibrosis or difficulties during the procedure. Their characteristics are listed in Table 2. Briefly, 5 patients had hypertension (50%), 4 patients underwent previous RP (40%), and one of them had adjuvant radiation therapy and is on androgen deprivation therapy, 2 patients had history of priapism (20%), 2 patients had dyslipidemia (20%), 1 patient had PD (10%), and 1 patient had previous transurethral resection of the prostate and peripheral neuropathy (10%).

**TABLE 2:**
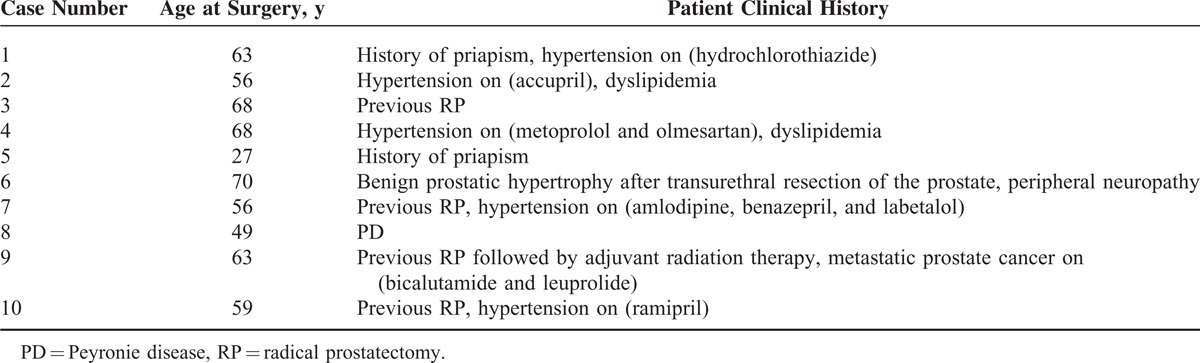
Control Patients’ Characteristics

### Histology

Tissue samples were prepared as we previously described.^10,11^ Briefly, penile specimens were fixed for 4 hours in a 0.002% picric acid and cold 2% formaldehyde 0.1 M phosphate buffer solution. They were then immersed overnight in a 30% sucrose buffer, placed in optimum cutting temperature compound (Sakura Finetek USA, Inc, Torrance, CA), and stored in −80°C until they were used. The specimens were then cut at 5 μm thickness, fixed into SuperFrost-plus charged slides (Fisher Scientific, Pittsburgh, PA), and allowed to air-dry for 5 minutes. We performed Masson's trichrome staining as previously described.^10^ Briefly, the previously prepared sections were immersed in warm (58°C) Bouin solution for 45 minutes and then rinsed. The samples were then stained with Weigert hematoxylin for 10 minutes and rinsed until only nuclei were visible. Staining then was done with Biebrich scarlet-acid fuchsin for 3 minutes, rinsed, and soaked in phosphomolybdic acid for 45 minutes. Then, we stained the sections in Aniline blue for 3 minutes, rinsed in distilled water for 2 minutes, then immersed them in 1% acetic acid for 2 minutes, and rinsed again in distilled water twice for 2 minutes. We dehydrated the sections in ethanol and allowed them to air-dry, and then mounted them. In order to prevent variations in staining, all samples were stained simultaneously. We captured the tissue section images using a digital camera, and used Plus 6.0 (Media Cybernetics, Inc, Bethesda, MD) to process them.

Lipid was visualized on frozen sections with the use of Oil Red O histochemical staining. After 2 minutes of incubation in propylene glycol, sections were stained in 0.5% Oil Red O in propylene glycol for 10 minutes at 60°C. Sections were differentiated in 85% propylene glycol, rinsed in distilled water, and stained in hematoxylin for 30 seconds.

For identifying the location of lipid deposition in the corpus cavernosum, we utilized LipidTOX and phalloidin double staining, which we previously described.^11^ Briefly, after washing with Phosphate buffered saline, the slides were incubated with Alexa Fluor 488 phalloidin (1:500, Invitrogen; Life Technologies, NY) followed by staining with LipidTOX neutral lipid stain (1:1000, Invitrogen; Life Technologies) at room temperature for 30 minutes. Nuclear staining was done by incubation with 4’,6-diamidino-2-phenylindole (DAPI, 1 μg/mL; Sigma-Aldrich, St. Louis, MO). For evaluation of cavernous lipid accumulation, 5 fields at 200× magnification on each tissue section were photographed. In each of these photographic recordings, the images were generated in green, red, and blue channels, and these single-color images were then superimposed to generate the multicolor figures. For quantification, the single-color images were analyzed with Image J software (National Institutes of Health, Bethesda, MD). To quantify lipid accumulation, the LipidTOX-stained area (in red) was measured and expressed as percentage of stained area per total area.

For image analysis, 5 randomly selected fields per tissue per patient were photographed and recorded using a Retiga Q Image digital still camera and ACT-1 software (Nikon Instruments Inc, Melville, NY).

### Statistical Analysis

The results were expressed as mean ± SD. Student *t* test was used for comparisons using Prism 5 software (GraphPad Software Inc, San Diego, CA). Values of *P* < 0.05 were considered statistically significant.

## RESULTS

**FIGURE 1:**
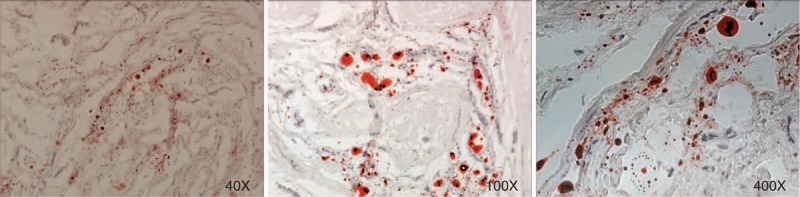
Oil Red O staining of corpus cavernosum from 3 different patients. The lipid droplets within the corpus cavernosum were conspicuous.

**FIGURE 2:**
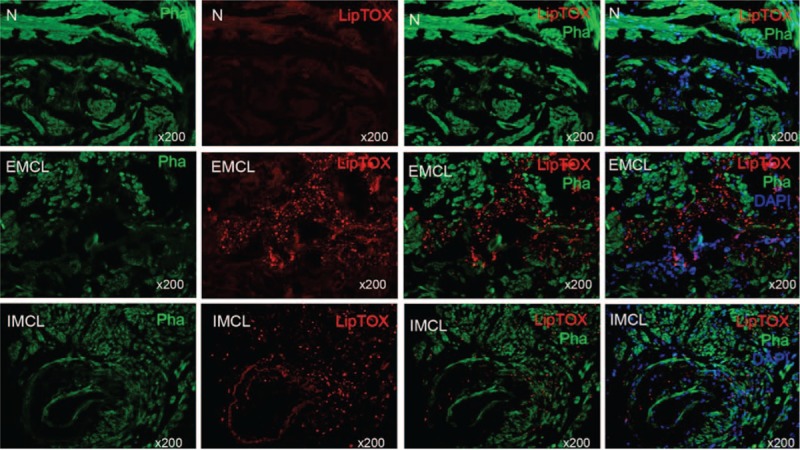
Lipid accumulation in human penis. In the control human penile tissue, there is no lipid (upper panel). The lipids (red, LipTOX) were distributed in 2 areas: without penile smooth muscle (middle panel) and with penile muscle (lower panel), represented as EMCL and IMCL, respectively (5.2 ± 1.3%) (original ×200). EMCL = extramyocellular lipid, IMCL = intramyocellular lipid.

**FIGURE 3:**
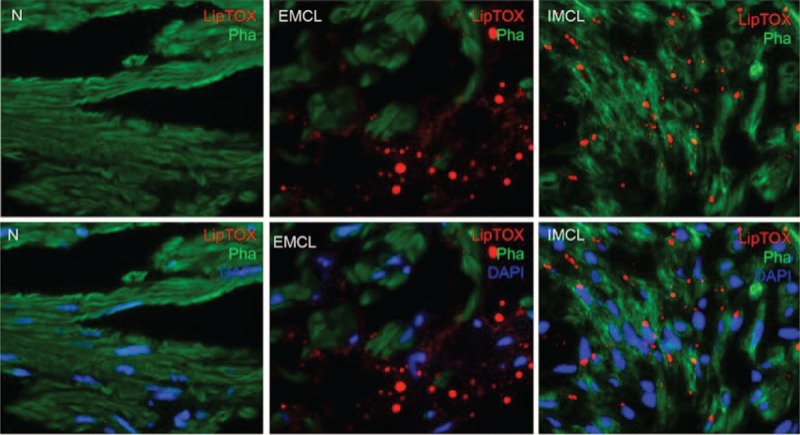
Localization of lipid within the corpus cavernosum. No lipids are seen in the control corpus cavernosum tissue (left panel). Lipid droplets were readily identified in our patients’ sections. Although most lipid droplets were EMCLs located in the interstitium outside the penile smooth muscle cells (EMCLs) (90%) (middle panel), some lipid droplets were IMCLs (10%) (right panel) (original ×800). EMCL = extramyocellular lipid, IMCL = intramyocellular lipid.

**FIGURE 4:**
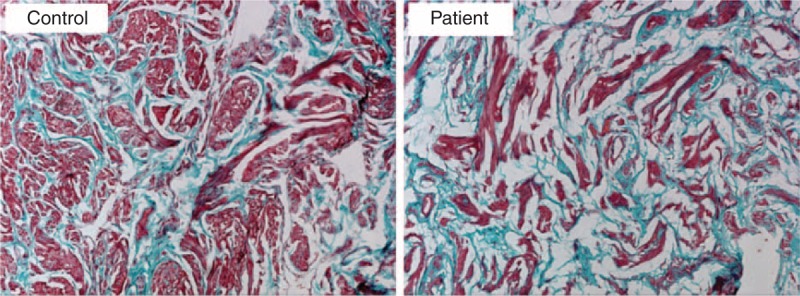
Smooth muscle contents in corpus cavernosum assayed with Masson's trichrome staining. Smooth muscle manifested as red, connective tissue was green. The amount of collagen is higher in the patient group with lipid accumulation (52.8 ± 9.6%) than in the control group (5.8 ± 0.67%) (*P* < 0.0001) (original ×100).

To our knowledge, no reports exist on the accumulation of lipids in human corpus cavernosum (whether normal or diseased). In our cohort of 9 patients, we demonstrate significant lipid content, which was visualized on Oil Red O staining. Figure 1 demonstrates the lipids in 3 different patients. Immunohistochemistry with LipidTOX and phalloidin double staining demonstrated accumulation of intramyocellular lipids (IMCLs) and extramyocellular lipids (EMCL) (5.2 ± 1.3%). Figure 2 compares patients with severe corporal fibrosis to control penile tissue. Further analysis of patients with severe corporal fibrosis demonstrated 90% EMCLs and 10% IMCLs (Figure 3). Masson's trichrome staining confirmed the presence of increased collagen in those 9 patients (52.8 ± 9.6%) when compared with control patients (5.8 ± 0.67%) (*P* < 0.0001) (Figure 4).

## DISCUSSION

There are no previously published reports of lipid accumulation in human corpus cavernosum, and therefore this case series is the first report about this finding. To our knowledge, there are also no reports of accumulation of fat in severe fibrotic states in other organs. Traish et al^12^ identified accumulation of fat-containing cells in the corpus cavernosum of castrated rabbits. They suggested that this is likely the result of androgen deficiency, and that androgen deficiency promotes differentiation of stromal progenitor cells into an adipogenic cell lineage instead of the anticipated myogenic cell lineage. They also suggested that the presence of fat-containing cells in the corpus cavernosum further impairs the veno-occlusive mechanism needed for erection, thus enhancing the negative effects of androgen deficiency. Another study showed that the administration of the endocrine disrupters bisphenol A and tetrachlorodibenzodioxin in intact rabbits resulted in fat accumulation in the corpora,^13,14^ although other simultaneous histological findings such as thickened tunica albuginea and increased content of smooth muscle are different between those described by Traish et al,^12,15^ suggesting possibly a different mechanism for fat accumulation.

Another possible explanation for lipid accumulation is the dedifferentiation of corpus smooth muscle cells into other phenotypes. Several reports in the literature have demonstrated the dedifferentiation of vascular smooth cells to other phenotypes.^16,17^ Corradi et al^18^ demonstrated that 5-α reductase inhibition resulted in prostate smooth muscle differentiation to other phenotypes. However, there are no studies directly examining trabecular smooth muscle dedifferentiation.

Our center is a tertiary care center for ED, and most of those patients represented challenging surgical cases that were referred to us, and we did not see them at their initial presentation. The primary surgeon in this study (T.F.L.) has been performing penile prosthesis surgery for over 30 years in a high-volume center. These 9 cases represented unusual cases in his practice in which a great difficulty was encountered during the procedure due to extensive fibrosis, and while carving out the corporal tissue it was noted to have a shiny whitish appearance suggestive of the presence of lipids, prompting us to examine these tissues specifically for fat. In order to validate these results, control corporal tissues (totaled 10 in number) from random ED patients going for penile prosthesis insertion were also examined for lipids, and they all were negative. The lipid accumulation in this study seems to be unrelated to metabolic syndrome because only 2 patients had DM, and 1 patient had dyslipidemia. Another fact to support that conclusion is that the majority (90%) of the lipids were EMCL, and it has been established previously that high IMCL rather than EMCL was associated with insulin resistance and metabolic syndrome.^19^ In addition, DM and dyslipidemia were well controlled for these patients. It remains however that this is a small sample to rule out an association with metabolic syndrome.

There are limitations in our study. Given that we were not the primary treating urologists for many of these patients, we did not see them at their initial presentation. Their serum testosterone levels were not measured; therefore, a link between androgen deficiency and lipid accumulation cannot be established. However, they did not experience fatigue, low libido, or other symptoms suggestive of hypogonadism to warrant their testosterone measurement clinically. Given the extensive fibrosis noted in those specimens, no biomarker profiling or endothelial dysfunction experiments were done. It remains however that this is the first report on the presence of lipid in human corpus tissue of patients with ED.

## CONCLUSIONS

This is the first report of lipid accumulation in the human corpus cavernosum. The exact mechanism of its accumulation is unknown and further research is needed. We encourage other urologists who encounter such a phenomenon during penile prosthesis insertion to examine these tissues for lipids and report their findings as well. It could also be beneficial to analyze this subset of corporal tissues for muscle biochemical markers and changes in the ultrastructure using electron microscopy, looking for evidence of smooth muscle dedifferentiation. Given the lack of reports of fat accumulation in other organs, it is our hope that other researchers might also investigate for lipid accumulation in different disorders associated with severe fibrotic states in order to understand this phenomenon.

## References

[1] Ayta IA McKinlay JB Krane RJ The likely worldwide increase in erectile dysfunction between 1995 and 2025 and some possible policy consequences. *BJU Int* 1999; 84:50–56. 10444124 10.1046/j.1464-410x.1999.00142.x

[2] NIH Consensus Conference. Impotence. NIH Consensus Development Panel on Impotence. *JAMA* 1993; 270:83–90. 8510302

[3] Shamloul R Ghanem H Erectile dysfunction. *Lancet* 2013; 381:153–165. 23040455 10.1016/S0140-6736(12)60520-0

[4] Lewis RW Fugl-Meyer KS Corona G Original articles: definitions/epidemiology/risk factors for sexual dysfunction. *J Sex Med* 2010; 7:1598–1607. 20388160 10.1111/j.1743-6109.2010.01778.x

[5] Zimmet P Alberti KGMM Shaw J Global and societal implications of the diabetes epidemic. *Nature* 2001; 414:872–877. 11742409 10.1038/414782a

[6] Gani J Radomski SB Management of erectile dysfunction in patients with sickle cell disease (CME). *J Sex Med* 2011; 8:2123–2127. 21791003 10.1111/j.1743-6109.2011.02399.x

[7] Carvalheira AA Pereira NM Maroco J Dropout in the treatment of erectile dysfunction with PDE5: a study on predictors and a qualitative analysis of reasons for discontinuation. *J Sex Med* 2012; 9:2361–2369. 22616766 10.1111/j.1743-6109.2012.02787.x

[8] Hsu G-L Hsieh C-H Wen H-S Anatomy of the human penis: the relationship of the architecture between skeletal and smooth muscles. *J Androl* 2004; 25:426–431. 15064322 10.1002/j.1939-4640.2004.tb02810.x

[9] Goldstein AM Padma-Nathan H The microarchitecture of the intracavernosal smooth muscle and the cavernosal fibrous skeleton. *J Urol* 1990; 144:1144–1146. 2231887 10.1016/s0022-5347(17)39677-5

[10] Lin G Wang G Banie L Treatment of stress urinary incontinence with adipose tissue-derived stem cells. *Cytotherapy* 2010; 12:88–95. 19878076 10.3109/14653240903350265 PMC2871776

[11] Lin G Qiu X Fandel TM Improved penile histology by phalloidin stain: circular and longitudinal cavernous smooth muscles, dual-endothelium arteries, and erectile dysfunction-associated changes. *Urology* 2011; 78:970.e1–970.e8. 21840580 10.1016/j.urology.2011.06.021 PMC3190031

[12] Traish AM Toselli P Jeong S-J Adipocyte accumulation in penile corpus cavernosum of the orchiectomized rabbit: a potential mechanism for veno-occlusive dysfunction in androgen deficiency. *J Androl* 2005; 26:242–248. 15713830 10.1002/j.1939-4640.2005.tb01091.x

[13] Moon DGLK Kim YW Park HS Effect of TCDD on corpus cavernosum histology and smooth muscle physiology. *Int J Impot Res* 2004; 16:224–230. 15184913 10.1038/sj.ijir.3901060

[14] Moon DGSD Kim YS Cheon J Bisphenol A inhibits penile erection via alteration of histology in the rabbit. *Int J Impot Res* 2001; 13:309–316. 11890520 10.1038/sj.ijir.3900734

[15] Traish AM Park K Dhir V Effects of castration and androgen replacement on erectile function in a rabbit model. *Endocrinology* 1999; 140:1861–1868. 10098525 10.1210/endo.140.4.6655

[16] Johnson JL van Eys GJ Angelini GD Injury induces dedifferentiation of smooth muscle cells and increased matrix-degrading metalloproteinase activity in human saphenous vein. *Arterioscler Thromb Vasc Biol* 2001; 21:1146–1151. 11451743 10.1161/hq0701.092106

[17] Rücker-Martin C Pecker F Godreau D Dedifferentiation of atrial myocytes during atrial fibrillation: role of fibroblast proliferation in vitro. *Cardiovasc Res* 2002; 55:38–52. 12062707 10.1016/s0008-6363(02)00338-3

[18] Corradi LS Carvalho HF Góes RM Inhibition of 5-α-reductase activity induces stromal remodeling and smooth muscle de-differentiation in adult gerbil ventral prostate. *Differentiation* 2004; 72:198–208. 15270776 10.1111/j.1432-0436.2004.07205004.x

[19] Godoy-Matos AF Bahia LR Domingues RC Rosiglitazone decreases intra- to extramyocellular fat ratio in obese non-diabetic adults with metabolic syndrome. *Diabet Med* 2010; 27:23–29. 20121885 10.1111/j.1464-5491.2009.02868.x

